# What Professor Bang’s Work Teaches1Read at the meeting of the Keystone Veterinary Medical Association, March 9, 1897.

**Published:** 1897-04

**Authors:** Leonard Pearson

**Affiliations:** Philadelphia


### WHAT PROFESSOR BANG'S WORK TEACHES.[1]

BY LEONARD PEARSON, B.SC., V.M.D.,
PHILADELPHIA.

PROFESSOR BANG, of the Imperial Veterinary School in Copenhagen, Denmark, is one of the greatest authorities on tuberculosis of cattle. His researches in this field have been published from time to time during the last fifteen years, and to him belongs the credit of having first discovered and called attention to many of the facts in connection with this disease that were previously unknown, but have since been repeatedly observed and re-demonstrated.

Denmark, Prof. Bang's country, is one of the smallest in Europe, but its inhabitants are thrifty, frugal people of a high order of intelligence and much conservatism. They depend largely, if not principally, upon the products of the dairy for their support, and although the country has a population of but 2,200,000, and an area of but little over 15,000 square miles, about one-third the area of Pennsylvania, there are in Denmark about 1,700,000 cattle (approximately the number in Pennsylvania). To illustrate the importance of the dairy it may be stated that in 1890 Denmark exported more than $22,000,000 worth of butter and more than $2,000,000 worth of cattle. This amounts to more than $10 for each inhabitant of the country.

It has been known for a long time that tuberculosis is a widespread disease among the cattle of Denmark, and various measures have been recommended and applied with a view to its suppression. From the above statistics it can readily be seen that the subject is one of great economic and national importance in Denmark, because it threatens the principal source of the country's wealth and prosperity. In 1892 an appropriation was made by the government for the support of investigations to be carried out by Prof. Bang for the purpose of determining the most practical means to be employed in restricting the ravages of this disease. The appro-

[1] Read at the meeting of the Keystone Veterinary Medical Association, March 9, 1897.

priations have been renewed from year to year and increased until at present they amount to about $30,000 per annum.

This government work has been conducted on such a large scale and in such a thoroughly careful way that the results are more instructive than those derived from similar experiments conducted elsewhere. One of Prof. Bang's recent publications was translated by the Hatch Experiment Station of the Massachusetts Agricultural College and published as Bulletin No. 41 in August, 1896, and this bulletin has been referred to and quoted from so frequently that Prof. Bang's name is now well known among readers of agricultural papers and those who take an interest in the discussions on tuberculosis of cattle. It is always possible, by quoting disconnected sentences and isolated paragraphs, to give an inaccurate and sometimes a very misleading impression as to the author's opinions and statements, and this has occurred in reference to the writings of Prof. Bang.

The writer has been familiar with the work of Prof. Bang for a long time, and has given his numerous publications careful study, which has been supplemented by conversation and correspondence with their author. The lessons that may be learned from Prof. Bang's writings and experiments are so important to us in this country at the present time that it seems well to formulate some of them and to attempt to express them without ambiguity. Among the most important are the following:

*Prevalence of Tuberculosis.* It had been known for a long time that tuberculosis prevailed extensively among Danish cattle, but the actual extent of the disease as revealed by the use of tuberculin was something of a surprise. In testing more than 53,000 cattle it was found that 38.5 per cent. of them were tuberculous to a greater or less degree. Moreover, it has been shown that the germs of tuberculosis are not omnipresent, because many herds are entirely free from all traces of this disease, and in some of these healthy herds the cows were large producers of the class.

*Degree of Contagiousness of Tuberculosis.* It has been clearly shown that the longer the disease exists in a herd the greater is its prevalence, leading to the belief, which is substantiated by other observations and experiments, that long-continued contact is necessary for the extensive prevalence of tuberculosis in the herd, and the longer such contact has existed the more extensive will be the spread of the malady. That some herds remain healthy notwithstanding the fact that they have been exhibited at cattle-shows and in public places, where they must inevitably have been exposed to the germs of tuberculosis for a short time, indicates that such exposure is not always dangerous. The infection in many herds has been traced to the introduction of a single diseased animal.

*The Infectiousness of Milk.* The milk from tuberculous cattle has been known to produce tuberculosis in calves and swine in very many cases, and also, strange to say, in such a comparatively immune animal as the horse. Attention is called to the dangers that accompany the use of skimmed milk from creameries, because if part of this milk is supplied by tuberculous cows, and the mixed product is returned to the farm, the disease may in that way be communicated to healthy herds; and common observation has proved that this is not rare. It is advised to heat all such skimmed milk to 185° F. before it is used. This destroys the tubercle bacilli.

*Heredity.* Tuberculosis is rarely inherited, and in all but the most exceptional instances the calves of tuberculous cows are sound when they are born, and if removed from contact with tuberculous animals and fed on milk from sound cows or milk that has been heated to 185° F. they will remain free from tuberculosis.

The matter of inherited predisposition to tuberculosis is considered, and some doubt is thrown upon its influence and even its existence. As there is no reason to assume "that a calf whose sire or dam suffers from tuberculosis accidentally acquired has thereby inherited a predisposition for tuberculosis which offers a more favorable nutritive soil for the development of the germs of tuberculosis, the predisposition, however great it may be, can play no practical part if infection is avoided."

*Tuberculin as a Diagnostic Agent.* The prevailing opinion as to the accuracy of tuberculin as a diagnostic agent is amply sustained, and it is established that the use of tuberculin furnishes by far the most accurate means of detecting tuberculosis and permitting the inspector to separate the healthy from the diseased cattle. It is made clear that the degree of reaction does not indicate the extent to which an animal is diseased, and that a high reaction may sometimes occur in an animal that is but slightly affected. It is also stated that from the degree of reaction conclusions as to the development of the disease must be drawn with great care. The reported failures to discover the lesions of tuberculosis in making post-mortem examinations upon animals that have been condemned by the use of tuberculin are incorrect in great part, since practically all of the tuberculous animals destroyed in European countries are killed in slaughter-houses. As their flesh is prepared for the market after inspection and under certain restrictions and exceptions, it is quite evident that all parts cannot be fully investigated, and doubt always accompanies a negative result.

Prof. Bang's personal experience, which is larger than that of any other veterinarian, has shown but three cases of typical reaction in which it was not possible for him to discover tuberculous deposits, and in one of these there was disease of a chronic and incurable character. It is stated, and this is well known to every one who has used tuberculin practically, that some severe cases do not respond to the test, but must be detected by a physical examination. Prof. Bang says that it is probably always possible to discover these causes by the usual clinical investigation, except where the disease has become stationary and is of slight development. These exceptions are minor and unimportant. Tuberculin is not to be relied upon implicitly as a diagnostic agent, but furnishes a method of diagnosis so incomparably superior to the methods previously employed that it is scarcely to be compared with them.

*Dangers Attending the Use of Tuberculin.* After an experience extending over 53,000 cases Prof. Bang is of the opinion that tuberculin is not injurious to healthy animals and that it cannot injure tuberculous animals, except by causing the disease to advance more rapidly, and that such "an acute development of tuberculosis as a result of tuberculin injection is to be feared only exceptionally and then in cases of advanced tuberculosis."

His final conclusion is "that we have now found that in tuberculin we possess if not an absolutely infallible, still an excellent means for recognizing tuberculosis, and that its application is not connected with any particular danger." It is also stated that "tuberculin has been employed upon a large scale for years, and still the demand from farmers constantly increases."

*The Use of Infected Cattle.* It is evident that if all tuberculous cattle in Denmark were destroyed, without compensation to their owners, the result would be widespread financial distress and ruin; and it is out of the question for the government to attempt to pay for all such cattle, because the amount required for this purpose would be beyond its resources. Therefore another method has, perforce, been adopted, viz., the retention of the animals that have reacted to the tuberculin test, and use them for breeding, for milk-production, and for the shambles. It has been found that under certain precautions the cattle can be so used with safety, and the saving is so great that a more radical method would under the circumstances be unjustifiable. But it must be observed that the tuberculous cattle are kept alive only in perfect isolation from healthy cattle. They are cared for separately and, when possible, by separate attendants. They are kept in separate buildings or in distinct and completely separated sections of a common barn. Their calves are removed the day after birth and are brought up on milk that has been heated to a point that will insure the destruction of tubercle bacilli. Their milk is used for the manufacture of butter (principally for export to England), but only after it has been heated to 185° F. In this way healthy herds are being developed from tuberculous ones, and, as the tuberculous cattle die or are killed for beef, the reacting division of the herd becomes smaller and smaller until finally it has disappeared, and thus tuberculosis is being allowed to die a natural death.

*The Curability of Tuberculosis.* Occasionally tuberculosis becomes latent after an animal has reacted to tuberculin; the subject improves in condition and fails to react upon subsequent injection, so that it may not be possible to confirm or re-establish the original diagnosis. This takes place most frequently in the cases that are less advanced; and some have thought that such cases might be cured. In order to throw light on this question Prof. Bang killed and made post-mortem examinations upon four animals of this sort, but found that they all had tuberculosis, and he says: "I therefore do not venture to draw from these observations the conclusion that the animals that failed to react one year after a typical reaction are to be regarded as cured. In many cases this conclusion would, perhaps, be justifiable; but, as it cannot always be the case, I consider it advisable to look upon animals that have once shown the typical reaction as suspicious and leave them in the reacting division."

*The Use of the Flesh of Tuberculous Animals.* Under proper inspection and certain restrictions and exceptions it seems to be quite admissible to use the flesh of some tuberculous animals without danger, and such is the practice not only in Denmark, but in all other European countries. Some carcasses are condemned outright and destroyed. Others are sterilized and sold as cooked meat, while still others are allowed to go upon the general market without restrictions or with the information that it is derived from tuberculous animals, so that those who purchase it may use it with especial care. These measures are generally regarded as sufficiently rigorous.

*It is Possible to Eradicate Tuberculosis.* Most important of all,

Prof. Bang has shown that by the use of tuberculin and measures based upon its use it is quite possible to eradicate tuberculosis in herds. This he has demonstrated on such a large scale in so many instances that there can be no doubt about it. Moreover, he has shown that by the employment of the Danish system the suppression of tuberculosis can be accomplished at comparatively small expense, and that the measures inaugurated in Denmark are constantly growing in popularity among live-stock owners.

His conclusion, as published in Bulletin No. 41, above referred to, is as follows: "The struggle against bovine tuberculosis must, of course, be of several years' duration. But it can and must be crowned with victory. In this struggle tuberculin has yielded us invaluable service. Only with the aid of this agent can we determine the actual extent of this disease. On the basis of the tuberculin investigations we are already in position to establish a rational plan of operation; and by this means alone can we retain the advantages gradually won; but the contest is well worth the pains. The conquest of bovine tuberculosis promises not only large economic profit, but also the annihilation of an important source of human tuberculosis."

